# The American Cyclopædia of Practical Medicine and Surgery

**Published:** 1835

**Authors:** 


### REVIEWS AND BIBLIOGRAPHICAL NOTICES.

---

### Art. III.—Cyclopædia of Practical Medicine.

*The American Cyclopœdia of Practical Medicine and Surgery, a Digest ef Medical Literature.* Edited by Isaac Hays, M. D.

The first volume of this comprehensive work, the most learned of any that as yet has issued from the American Medical Press, is completed, and the first number of the second volume is now before us. We cheerfully commend it to our readers, as a rich repository of medical science, collected from every available source, and, in general, digested and arranged with care and sound professional common sense. As a specimen of the second volume, we lay before our readers the following extract on the sedative effects of the Tartrate of Antimony.

"*Tartar emetic as a contro-stimulant.*—Rasori, professor of clinical medicine at Milan, published in 1800 his views on the therapeutic action of tartar emetic in large doses, in controlling inflammatory excitement in certain phlegmasial diseases, particularly peripneumony. He denoted the condition of high excitement characterizing these diseases, as the *diathesis of stimulus;* and hence he called tartar emetic in large doses, as administered by him, a *contro-stimulant.* Peschier of Geneva, Laennec, and many other practitioners confirmed the general accuracy of his results; and no one at present calls in doubt the curative power of tartar emetic, in large doses, in the treatment of peripneumony. It has also been recommended, in the same doses, in several other diseases; such as hemoptysis, pleurisy, articular rheuma[illegible]sm, apoplexy, traumatic tetanus, encephalitis, phlebitis, puerperal [illegible]ritonitis, &c. Numerous observations have incontestably proved, a[illegible] tartar emetic, given by the method of Rasori, will control most th[illegible] inflammations; but it is doubted by many physicians, whether [illegible]actice is safe, and whether it does not infinitely more harm to the dig[illegible]ve organs, than good in subduing local inflammation in other par[illegible] The particular mode in which it acts, according to the views of R[illegible], will be detailed in another article: (see *Contro-stimulant.*)

Some have supposed its mode of action, when given by the method of Rasori, to be sedative; but its mere power to diminish inflammatory excitement by no means proves it to be so. We believe, with Broussais, that it acts as a powerful revulsive on the stomach; but, without entering, in detail, upon the theory of its operation, we shall use the term *contro-stimulant*, to express the mode of action, whatever it may be, of tartar emetic, when given in the manner recommended by Rasori.

"According to this last writer, tartar emetic, in large doses, so as to produce its contro-stimulant operation, is borne only in the sthenic or inflammatory condition of the organism, or, as he expresses it, during the existence of the diathesis of stimulus. This explanation of the tolerance of the remedy, in doses which under other circumstances would prove poisonous, is consonant with pathological principles; but while Trousseau admits that the remedy is never better supported than when the inflammatory symptoms are most intense, he nevertheless asserts that he has seen tolerance to be perfectly established in patients excessively weak. He, therefore, denies, that the inflammatory condition is essential to the tolerance of antimonials, and explains the fact that they are not well borne in health, by the circumstance, that a rigorous diet, an essential condition of tolerance, is not adopted. The different view of Trousseau on this subject may be explained by the fact that he prefers the use of the oxides, in obtaining the contro-stimulant effect of antimony, preparations which produce incomparably less irritation and disturbance of the system than tartar emetic.

"The chief disease in which Rasori has given tartar emetic as a contro-stimulant is *peripneumony*. His principles of treatment may be summed up under the following heads: 1. To treat the disease throughout by tartar emetic; 2. To adopt this medicine as the principal, and sometimes the only remedy in the disease; 3. To diminish by its use the number of bleedings, or to do away the necessity of this evacuation altogether; 4. To give the remedy in doses which formerly the boldest practioners never thought of employing; amounting to one or more drachms in the twenty-four hours, and to several ounces in the course of the disease, without producing either vomiting or purging.

"Rasori, and the Italian physicians, in treating peripneumony, generally give at first from ten grains to a scruple of tartar emetic, and increase the dose gradually to one or several drachms in the twenty-four hours, in proportion as the tolerance of the remedy is gradually established. Laennec, who with M. Kapeler, were among the first to call attention to the method of Rasori in France, adopted it with slight modifications. When a patient with peripneumony would bear [illegible] bleeding, Laennec began the treatment by drawing from eight to si[illegible]teen ounces of blood; and rarely repeated the venesection, unless [illegible]n individuals attacked with diseases of the heart, or threatened with [illegible]poplexy or some other sanguineous congestion. He states, however[illegible]hat he has frequently and rapidly cured intense peripneumonies [illegible]hout recourse to bleeding, though generally he resorted to it. Imm[illegible]ately after the bleeding, he gave a grain of tartar emetic in two flu[illegible] ounces and a half of sweetened aromatic water, and repeated the dose every two hours, until six had been taken; after which the patient was allowed to rest for seven or eight hours, if the symptoms were not urgent, and a disposition to sleep was manifested. But if the disease was already advanced, the oppression great, the head seized, and both lungs affected, or if one of them was invaded throughout, he was in the habit of continuing the tartar emetic without interruption, until the symptoms were mitigated, and the stethoscope indicated an improvement. When, however, the agravating circumstances, above mentioned, were all united in the same case, his practice was to increase the dose of the antimonial to a grain and a half, two grains, or even two grains, and a half, but given always in the same amount of vehicle.

"Laennec remarks, that some pneumonic patients support tartar emetic, administered in this manner, without vomiting or purging. A majority, however, vomit several times, and have five or six stools the first day; but afterwards they experience but moderate evacuations, and often none at all. After the tolerance of the medicine has been established, it is asserted by Laennec, that the patients often become constipated, so as to require the use of purgative enemata.

"In describing this mode of practice, Laennec proceeds to remark, that, when evacuations continue to the second day, or when there is reason to believe that the tartar emetic will be supported with difficulty, he adds to the six doses to be taken in twenty-four hours, one or two ounces of the syrup of diacodion, (syrup of poppies,) an addition which he admits is contrary to the theoretic views of Rasori, but which his experience has proved to be useful. He declares further that, in general, the effect of the tartar emetic is never more rapid than when it causes no evacuation whatever; though sometimes its good effects are accompanied by a general sweat. Though he considers frequent vomiting and abundant purging to be feared, on account of the weakness, and the irritation of the alimentary canal which they produce, yet he avers that he has effected remarkable cures, where the evacuations were very abundant. 'I have,' says he, 'very rarely met with pneumonic patients who could not support tartar emetic, and such cases occurred in my first trials; so that this inconvenience is attributable, perhaps, to the inexperience and want of confidence of the physician, rather than to the method itself. In many cases, where a patient would support, only tolerably, six grains of tartar emetic with syrup of diacodion, one day, I have given him the next day nine grains with perfect tolerance. At the end of twenty-four or forty-eight hours at most, and often at the end of two or three hours, a marked melioration of all the symptoms is obtained. Sometimes, even, a patient who appears to be doomed to certain death, is, at the end of a few hours, out of danger, without having experienced any crisis or evacuation, or any other notable change than a progressive and rapid improvement in all the symptoms; and the exploration of the chest explains the reason of the sudden change, by detecting all the signs of resolution.'

"Laennec states that effects equally striking may be obtained at all stages of the disease, even when a great part of the lung is infiltrated with pus!

"From the moment an improvement takes place, Laennec conceives that the medicine may be continued with the certain result of completing the resolution, without inducing fresh commotions; a circumstance in which mainly consists the great practical advantage of tartar emetic over blood-letting as a remedy. By bleeding, he admits that we almost always obtain a diminution of fever, of oppression, and of bloody expectoration; but at the end of some hours, these symptoms again increase; and this often happens five or six times, after the employment of as many bleedings. Now Laennec affirms that he had never seen similar relapses under the use of tartar emetic; and that when this antimonial was employed the weakness during convalescence was never so protracted, or excessive, as when the patient was treated by repeated bleedings.

"We by no means subscribe to the accuracy of all the above views of Laennec, in relation to the tartar emetic treatment in peripneumony; but though his assertions may have been too general on some points, and his plan of practice too little regulated by the state of the alimentary canal, still the opinions of so distinguished an authority must always be viewed as important, and entitled to great respect.

"M. Peschier of Geneva, advocates the contro-stimulant use of tartar emetic in peripneumony, and in fluxions of the chest generally; but his mode of using it appears by no means precise, and not at all regulated by the state of the system, When a determination to the skin was manifested, he added nitric, muriatic, or acetic ether to the antimonial; and when there existed dysuria, and dry heat of the skin, he conjoined nitre. He generally began the treatment with six grains in the twenty-four hours, and increased the quantity three grains daily, until the dose reached twelve or fifteen grains, a quantity which he did not exceed, as this was found sufficient. In cases in which, from the great weakness of the patient, he was induced to direct so small a dose as a grain, or a grain and a half, it produced fatiguing effects, without any curative result. In none of the cases which he treated, was blood-letting, either general or local, employed, but blisters were occasionally resorted to.

"Without implicitly relying on the results obtained by Rasori, Laennec, and M. Peschier, from the use of tartar emetic as a contro-stimulant, their general tenour is sufficiently coincident to satisfy the reader of the remarkable controlling power of this remedy, when thus employed, over acute pneumonic inflammation. Still the question arises how far the practice is a safe one, and how far it may be used to replace the ordinary curative means, of general and local blood-letting, blistering, antiphlogistic medicines, demulcents, and anodynes. In determining this question, the more recent results deduced by M. Rayer from careful observations made on a number of pneumonic patients at the hospital La Charité, will have an important bearing, from the impartial manner in which they appear to have been conducted. M. Rayer arranges his observations under the three heads, of effects on the digestive organs, on the organs of respiration, and on the circulation and blood.

"Effects on the digestive organs of pneumonic patients.—1. Tartar emetic, dissolved in a small quantity of sweetened vehicle, produces vomiting less easily than when it is dissolved in pure water, and assisted by the administration of warm and nauseating drinks.

"2. The tolerance of the remedy was obtained in a *few* patients from the first day, without any apparent reason for its want of action on the stomach or bowels. Now this tolerance is affirmed to take place in a *great number* of patients by Rasori, and in *many* by Laennec.

"3. The majority of the patients vomit on the first day, after having taken the first doses. They experience at first a general uneasiness with disposition to vomit, afterwards paleness, and contraction of the volume of the body. After vomiting, reaction and heat return in the course of half an hour or an hour. During the interval, the patient is pale, with a small concentrated pulse, and yet he declares himself to be better.

"4. Some patients, from irritability of the digestive organs, support the contro-stimulant method with difficulty, being affected with violent vomitings, colics, and twistings of the bowels.

"5. Tolerance is established more freely and permanently in relation to the stomach than to the bowels; many patients experiencing purging without vomiting after the first days.

"6. In patients with healthy stomachs, the vomitings and purgings were not preceded or followed by pains in the abdomen, and were not accompanied by any pain except that attendant on the act of vomiting. The next day the stomach and bowels were rarely affected with pain.

"7. The alimentary canal soon loses its power of tolerance; for if the treatment be discontinued for a day or two, the same doses, which had ceased to produce vomiting and purging, and even smaller doses, will reproduce them.

"8. Tartar emetic may be given for many days together in very large doses to some pneumonic patients, without producing any evident inflammation of the alimentary canal; but this is not always the case.

"9. If on the one hand, the generality of physicians have exaggerated the irritant properties of tartar emetic; on the other, the *contro-stimulists* have been guilty of an opposite exaggeration, far more dangerous.

"10. When it is wished to establish a tolerance of the remedy, it is better to augment the dose gradually, or even to diminish it, rather than to employ opiates, which gave rise to a factitious tolerance, masking the effects of the antimonial on the digestive organs.

"11. Tartar emetic, administered in large doses, produces sometimes inflammation of the stomach and bowels; but these artificial inflammations are, in general, less serious and obstinate than those generated without appreciable causes; and when they are not kept up by the too long-continued action of the remedy, nor aggravated by a previous affection of the stomach, they yield, with sufficient readiness, on the suspension of the remedy, or to the use of a few local bleedings.

"12. During the convalescence of pneumonic patients, in whom resolution is progressing, tartar emetic, in the dose of five or six grains, appears to excite the feeling of hunger. After tolerance has been established, digestion does not appear to be deranged by the remedy, when administered three hours before or after a meal. It may be proper to remark, that many of M. Rayer's patients were on half-diet.

"13. In this paragraph, M. Rayer reports the post-mortem appearances observed in the few pneumonic cases which he lost. These have been detailed in a preceding part of this article. (See p. 54.)

"Effects on the organ of respiration.—1. The effects of tartar emetic, even in large doses, upon the organs of respiration, vary according to the intensity of the peripneumony, and to the disturbance, more or less serious, which it impresses on the digestive organs. M. Rayer has seen peripneumonies disappear in forty-eight hours after repeated vomitings and purgings by tartar emetic. He has observed other cases in which the disease progressed, although there was a marked tendency to tolerance. In a considerable number of cases, the cough and bloody expectoration diminished in an evident manner, and in the following days the stethoscope detected the progressive march of resolution.

"2. By means of this treatment, and by the use solely of tartar emetic, many peripneumonies, both simple and double, were completely cured, and in as short a time as when treated by blood-letting.

"3. The good effects of tartar emetic are never more evident, according to the opinion of M. Rayer, and in opposition to that of Rasori, than when it produces abundant evacuations. Indeed, the absence of tolerance, signalized by Laennec as one of the contra-indications of tartar emetic, is, according to M. Rayer, on the contrary, oftener one of the conditions most favourable to its employment, provided the stomach be not inflamed.

"4. The first days of the use of tartar emetic in recent peripneumonies are distinguished by a decided melioration of the disease; but this improvement becomes afterwards less and less evident, from the tolerance of the remedy, and from the fact that those diseased conditions disappear first which are the least fixed.

"5. The property attributed to tartar emetic of augmenting the energy of interstitial absorption, so as to resolve pulmonary hepatizations after bleedings have lost their influence, as appeared to M. Rayer to be a very questionable one in the majority of cases.

"6. The quantity of tartar emetic necessary to effect the cure of peripneumony varies from several drachms to one or more ounces, according to the extent of the pulmonary inflammation, and the duration of the disease before the treatment is commenced.

"Effects on the circulation and blood.—When tolerance is established, the action of tartar emetic on the circulation is scarcely appreciable; on the contrary, when vomiting is about to take place, and during the existence of colicky pains, the pulse becomes concentrated. With regard to the buffy appearance so uniformily exhibited by the blood in pneumonia, M. Rayer ascertained that this appearance was not modified by the tartar emetic treatment.

"M. Rayer sums up his results by declaring that, in his opinion, the treatment of peripneumony by tartar emetic, as an *exclusive method*, is inferior, in a majority of cases, to the treatment of blood-letting.

"Before resorting to the method of Rasori, Rayer further remarks, it is necessary to be satisfied as to the condition of the digestive organs. It must not be forgotten that many individuals have latent affections of the stomach, and that certain results of chronic gastritis in old persons, such as thinning and softening of the stomach, are always aggravated by tartar emetic. Moreover, this method is hurtful, when the pneumonia is complicated with gastritis. But when the integrity of the digestive organs is well ascertained, it will be proper, according to M. Rayer, to employ tartar emetic and bleeding concurrently in the treatment of peripneumony. This combined method he conceives to be preferable to all others at the commencement of pulmonary inflammations; but instead of *aiming to produce tolerance*, it is preferable, according to him, to *obtain abundant evacuations*. Six, eight, ten, or fifteen grains of the antimonial are considered sufficient by him to produce this result; it being unnecessary to increase the quantity daily until the dose reaches a drachm. He, accordingly, characterizes it as a blameable temerity to imitate Rasori, who has often given the almost incredible quantity of an ounce in the course of a day. Finally, M. Rayer recommends, that in proportion as resolution progresses, the dose of the tartar emetic should be diminished, having care always to continue it for some time after the disappearance of the crepitant rhonchus.

"We have presented thus fully the results of M. Rayer, on account of their intrinsic value, and of the precision with which they are reported. But we cannot admit all his therapeutic views, and particularly that one which asserts that the contro-stimulant action of tartar emetic is never more marked than when it produces copious evacuations, and that the establishment of tolerance is rather detrimental than useful to that action. The great authority of Laennec is opposed to it, and the observations of Rasori, M. Peschier, M. Trousseau, and of many other practitioners, establish a diametrically opposite conclusion, We admit the concurrent benefit of blood-letting, and cannot conceive how it could interfere with the curative operation of the tartar emetic. Dance agrees, on this point, with M. Rayer; but M. Trousseau states emphatically, as the result of his experience, that blood-letting in peripneumony, so far from assisting, impedes the operation of antimony. He asserts that resolution is not completed for a long time, when bleeding is employed; while it is never more rapid than under the sole influence of antimony. In short, he declares it as the chief merit of the cure of peripneumony by antimony, that there is *no convalescence.* Patients, according to him, are sometimes brought, in the course of a few days, from the brink of the grave, to a state of apparent health so satisfactory, that, without the indications of the stethoscope, it would be impossible to believe that there had existed a dangerous peripneumony. Of fifty-eight pneumonic patients treated by M. Trousseau, none of whom were bled in the hospital, and five only before admission, but two died. The cases most rapidly cured by the antimony were precisely those in which the disease was most recent, the fever most vehement, the pulse most full and vibrating, the skin hottest, the oppression greatest, the local pain most acute, and the expectoration most bloody.

"Tartar emetic as a contro-stimulant has been given in *hæmoptysis* and *pleurisy*, but with doubtful advantage. Laennec contended that it had the power of controlling the inflammatory action in the latter disease; but M. Trousseau reports that, in ten cases in which he tried antimony in acute pleurisy, no reduction of the diseased excitement was obtained.

"According to Laennec, acute *articular rheumatism*, is, after peripneumony, the inflammatory disease in which tartar emetic in large doses appeared to him to be the most efficacious. The mean duration of rheumatism, treated by this curative plan, was seven or eight days; while, by the ordinary method of treatment by bleeding, &c., it is known to last from one to two months. The medicine, however, is less efficacious, where muscular and articular rheumatism are combined. Laennec sometimes though rarely observed relapses after articular rheumatism, when the medicine had not been discontinued; and he was obliged, in two cases only, to interrupt its use, because tolerance could not be established. Of thirteen rheumatic cases treated by Laennec, tartar emetic proved highly useful in eight, without effect in two, hurtful in one, and of doubtful utility in two.

"M. Rayer dissents from Laennec as to the value of the contro-stimulant practice by tartar emetic in rheumatism; and rests his dissent on the variable character and duration of this disease, which forbids the adoption of any opinion as rigorously exact, in favour of any particular plan of treatment. Upon the whole, he comes to the conclusion, in opposition to Laennec, that the plan by blood-letting, and by revulsives applied to the skin, is preferable to that by tartar emetic in large doses. Besides, tartar emetic is strongly contra-indicated in cases in which the rheumatic disease is partly seated in the digestive organs, or likely to be translated to them. Rayer concludes by remarking, 'that the employment of tartar emetic in large doses in rheumatism is more rarely indicated than in pneumonia; for the danger to life, in the latter disease, is such as to make it admissible to produce a momentary or sustained revulsion on the stomach and intestines; while it is by no means demonstrated that this is equally true with regard to rheumatism.'

"M. Trousseau reports his experience with the use of large doses of tartar emetic in acute articular rheumatism, and is of opinion that, when of service, it does good by operating as an emetic and cathartic, rather than as a contro-stimulant. Its good effects, however, are by no means so constant as in pneumonia; and so far from acting on the principle of tolerance, it is never so useful, according to him, in acute rheumatism, as when it produces vomiting and hypercatharsis. It is on this account that M. Trousseau denies to the antimonials the possession of any special curative power in this disease, and attributes their efficacy exclusively to their action as evacuants.

"Laennec and other practitioners have employed contro-stimulant doses of tartar emetic in *apoplexy*. Of eleven cases treated on this plan, six were cured; but as blood-letting was used at the same time, it is impossible to determine the precise curative value of the tartar emetic. In some cases of apoplexy, Laennec has gradually carried the dose to a drachm and a half; but it should be recollected that, in consequence of the lesion of the nervous system which exists, the tolerance of the antimonial in this disease is often apparent, not real, and, therefore, cannot be held as proof of the safety of the stomach.

"MM. Delpech and Lallemand have recently extended the contro-stimulant use of tartar emetic to the prevention and cure of *tetanus* from *traumatic lesions*. Their results were made known by M. J. Franc, one of their pupils, in a memoir published in 1834, from which it appears that the exhibition or tartar emetic in large doses prevents the accidents which follow those lesions; and, when these accidents have already taken place, forms the most efficacious means of treating them. By this treatment, M. Lallemand has cured, in many cases, a slight and commencing *encephalitis*, the consequence of traumatic lesions. The dose of the antimonial which he employs in the twenty-four hours, is generally eight grains, associated with the syrup of poppies. Delpech, however, gives the remedy in simple aqueous solution without any addition.

"M. Trousseau reports several cases of phlebitis and puerperal peritonitis, successfully treated by tartar emetic and other antimonials, used as contro-stimulants.

"In concluding our remarks on the contro-stimulant use of tartar emetic, it is hardly necessary to add that its employment in this way is strongly contra-indicated in inflammation of the stomach and bowels. M. Fabre has reported a case of combined bronchitis and gastritis, in which the administration of twelve grains of tartar emetic was followed by bloody vomitings, convulsions, and delirium. M. Vacquie has recorded a case of acute gastro-peritonitis, made to terminate in gangrene, and M. Barbier, two cases of inflammation of the stomach, exasperated and rendered rapidly mortal, by the use of large doses of this antimonial. Too much caution, therefore, cannot be exercised in its administration in diseases in which the alimentary canal is implicated."

Our limits permit but one other extract, we shall make it from an able and elaborate article on the physiology and diseases of the anus; selecting what relates to the atony of that part.

"*Atony of the Anus.* In consequence of frequent and undue dilatation of the anus, the two sphincters sometimes cease to contract with sufficient force, and the canal becomes permanently enlarged. The same result also happens occasionally from paralysis consequent upon concussions of the spine, and the enlargement may then continue after the spine has recovered from the effects of the injury. Among the most fertile causes of the worst forms of this affection, may be ranked the indulgence in unnatural crimes, fortunately little known in this country, but which the crowded condition of those schools of vice, our public prisons and alms-houses, are rapidly introducing amongst us.

"Slight shades of dilatation from atony of the sphincters are very common among men of sedentary habits, more particularly such as are esteemed good livers; though they are seldom made a ground of complaint, and have received much less attention than they deserve. The immediate consequences of this loss of tone are, a slight irritation of the teguments, and an increased secretion of the mucus of the canal, which sometimes produce serious inconvenience, although unattended by pain. As ulterior results, this morbid condition may determine the development of many of the more serious diseases of the part, under the action of agents which would prove harmless in a healthy condition of the anus; and these diseases may recur after a cure has been accomplished, if the atony be neglected. The complaint, in such cases, yields almost invariably to a regimen and treatment calculated to regulate the habitual action of the bowels, aided by that most powerful but much neglected remedy in irritations of this part, the local application of cold. The best mode of applying this remedy is to throw a continued stream of cold water upon the anus; and it is much to be regretted that the apparatus for what is termed the *douche ascendante* is so seldom employed in this country. Cold ablutions, however applied, are much less efficacious.

"The worst forms of the complaint, originating from the causes already enumerated, or from tumours, or former operations on the part, are attended by permanent stretching of the teguments themselves, remaining after the sphincters have in a great degree recovered their tone. Such cases yield only to the removal of the cause, to the use of astringents, or, in the most obstinate, to the excision of some of the redundant folds of the skin. (See *Prolapsus Ani.* § 5.)

"If the affection is the result of vicious habits, says Velpeau, the anus is strongly excavated, like a funnel; in other cases, it is salient, depressed, or irregular, but never infundibuliform. Involuntary stools and prolapsus ani are often occasioned by this kind of enlargement. When the causes which originally produced it are incurable, as in some cases of tumour and paralysis, the case is beyond the reach of art."

